# Delirium in critically ill children: a retrospective pre- and post-cohort study on the introduction of delirium screening in a paediatric intensive care unit

**DOI:** 10.1007/s11096-025-01887-2

**Published:** 2025-05-07

**Authors:** Diarmaid Semple, Fiona Boland, Cormac V. Breatnach, Moninne M. Howlett, Judith D. Strawbridge, John C. Hayden

**Affiliations:** 1 Children’s Health Ireland Crumlin, Dublin, Ireland; 2 School of Pharmacy and Biomolecular Sciences, Royal College of Surgeons in Ireland, Dublin, Ireland; 3 Data Science Centre, School of Population Health, Royal College of Surgeons in Ireland, Dublin, Ireland; 4 School of Medicine, The University of Dublin, Trinity College, Dublin, Ireland

**Keywords:** Paediatric delirium, Paediatric critical care, Analgesia and sedation, Pharmacotherapy

## Abstract

**Background:**

Paediatric delirium is a neuropsychiatric disorder with disrupted cerebral functioning due to underlying disease and/or critical care treatment. It has been reported in up to one third of paediatric intensive care admissions, with hypoactive delirium most prevalent in children.

**Aim:**

The aim of this study was to assess whether the introduction of delirium screening was associated with a change in the pharmacotherapy exposure and clinical outcomes.

**Method:**

A retrospective pre and post cohort study of all admissions > 48 h who required mechanical ventilation between 11th March 2019 and 11th March 2021. Cohort 1 (11th March 2019–11th March 2020) prior to the introduction of delirium screening and cohort 2 (12th March 2020–12th March 2021) after delirium screening. Patients < 3 months old, who were never mechanically ventilated, admitted <48 h, continuously receiving neuromuscular blockade or deeply sedated were not included. A multivariate model was created to compare pharmacotherapy use before and after implementation of delirium screening.

**Results:**

Two thousand and thirty-four patient encounters were identified with 588 meeting the inclusion criteria (364 cohort 1 and 224 cohort 2). There was a reduction in usage of infusions of morphine (decrease in doses of 18% *p* < 0.05) and midazolam (50% reduction in patients receiving *p* < 0.05), after screening commenced. Chloral hydrate use was unchanged however cohort 2 received lower daily doses (*p* < 0.05). Clonidine infusion use increased for cohort 2 (16% v 28% *p* < 0.05), with lower daily doses (23 v 13 µg/kg/day *p* < 0.05). Positive clinical outcomes such as decreased duration of mechanical ventilation, length of stay and out of range sedation and withdrawal scores were also observed.

**Conclusion:**

Introduction of a paediatric delirium care bundle including screening tool and associated education was associated with decrease in exposure to modifiable pharmacotherapy risk factors for the development of paediatric delirium. These findings should be further evaluated in future interventional studies.

**Supplementary Information:**

The online version contains supplementary material available at 10.1007/s11096-025-01887-2.

## Impact statements

- Delirium screening can help reduce exposure to analgesia and sedation, which are modifiable risk factors for paediatric delirium.
- Delirium screening can result in changes to prescribing patterns whilst maintaining adequate analgesia and sedation for critically ill children.
- This study shows you can optimise pharmacotherapy without compromising patient care or comfort.

## Introduction

Paediatric delirium (PD) is a neuropsychiatric disorder with disrupted cerebral functioning due to underlying disease and/or critical care treatment [1–3]. Paediatric delirium has been reported in up to one third of paediatric intensive care unit (PICU) admissions, with hypoactive delirium most prevalent. It has been associated with prolonged mechanical ventilation times, length of stay, higher costs, and a reduction in quality of life [4]. The European Society of Paediatric and Neonatal Intensive Care (ESPNIC), the Society of Critical Care Medicine and World Federation of Paediatric Intensive and Critical Care Societies have advised routine screening of children of all ages for ‘ICU delirium’ using a validated tool [5, 6]. A 2014 international survey of intensivists reported that delirium screening was not practiced in 71% of respondents' PICUs, and only routinely practiced in 2% [7]. In a 2015 survey of paediatric cardiac intensive care clinicians, 86% of respondents were not satisfied with management and screening of delirium, with 7% reporting routine screening for paediatric delirium [8]. Barriers to screening have been identified to include a lack of education, physician engagement, and concerns regarding time involved or complicated nature of screening tools [9].The development of paediatric delirium is associated with modifiable and non-modifiable risk factors. Modifiable risk factors include opioid and benzodiazepine exposure and cumulative dose, number of sedative classes used, use of restraints and mechanical ventilation (MV). Non-modifiable risk factors include age, developmental delay and severity of illness [4, 10].

As part of a Children’s Health Ireland (CHI) quality improvement initiative (QI), a multidisciplinary group composed of physiotherapists, clinical nurse facilitators (CNF), clinical pharmacist and medical consultants introduced the Sophia-Observation Withdrawal Symptoms Paediatric Delirium scale (SOS-PD) to both Children’s Health Ireland (CHI) at Temple Street (formerly known as Temple Street Children’s University Hospital) and CHI at Crumlin (formerly known as Our Lady’s Children’s Hospital, Crumlin) PICUs. The SOS-PD tool is a screening tool based on the Sophia Observation withdrawal Score (SOS) with an additional seven items validated to detect paediatric delirium [13]. A score of ≥ 4 or reports of hallucinations indicates the presence of paediatric delirium [12]. At the time of implementation CHI Crumlin used SOS to detect withdrawal (positive score indicated by result ≥ 4) and the COMFORT behavioural (COMFORT-B) tool with a numerical rating scale between 0 and 10 (NRS) to guide analgesia and sedation management [14, 15].

Simone et al. have reported compliance rates of >95% for paediatric delirium screening after the introduction of a bundle that included; PD education; the introduction of a PD screening instrument; intervention and treatment algorithm and sedation and early mobility protocols [16]. Previous published studies have not addressed the impact of the implementation of paediatric delirium screening on the exposure to modifiable risk factors, particularly pharmacotherapy and resultant clinical outcomes. This study sought to address these gaps.

### Aim

Our hypothesis was that the introduction of paediatric delirium education, a delirium screening tool (SOS-PD), a process map and ‘Delirium Algorithm’ would reduce the exposure of patients to modifiable pharmacotherapy risk factors such as opioids and benzodiazepines.

The objective of this study was to assess whether the introduction of delirium screening into the PICU was associated with a change in pharmacotherapy exposure and clinical outcomes.

### Ethics approval

The Children’s Health Ireland Research and Ethics Office determined the study met the requirements for clinical audit and provided an exemption from a full ethics application.

## Method

### Study design

We conducted a retrospective cohort study of all patients admitted to the PICU of CHI Crumlin, Dublin, Ireland between 11th March 2019 and 12th March 2021 who met our eligibility criteria.

### Setting

The PICU in CHI Crumlin is Ireland’s largest PICU with 23 Beds and approximately 1000 annual admissions [17].

### Intervention

The Delirium QI team prepared a bundle of resources consisting of: instructions and videos publicly available from Erasmus MC-Sophia Children’s Hospital [18]; presentations (tailored to the time available and format of activity) on theory; paper and electronic ‘PD-SOS Process Map’ (Supplementary Fig. 1); a ‘Delirium Algorithm’ (Supplementary Fig. 2) and a paper copy of the SOS-PD score was placed in the bed-side folder of all beds; supervised scoring using real-world patient care scenarios or video resources [11, 12, 18]. The resource material was saved on a shared folder for access and reference by staff during their working shift. The QI team delivered a mixture of individual training including mock scoring at bedspaces and in small groups with nurses, doctors and healthcare assistants, longer format presentations and mock screening sessions with mostly nursing staff, tailored training to doctors as part of their medical education sessions were provided, awareness posters, screensavers, culminating in an ‘official launch’ on World Delirium Awareness Day (WDAD) with posters, quizzes and ‘drop-in’ event for staff. Local training practice for nursing is that 80% of staff are educated prior to commencement of a policy.

Ability to document SOS-PD scoring was added to the PICU clinical information management system in CHI Crumlin in Oct 2019. Staff training was completed and scoring formally commenced as PICU policy on WDAD 11th March 2020. Local policy is to screen all admissions, regardless of age after 24 h. This is despite the SOS-PD tool validation only in patients > 3 months old and admissions > 48 h. This decision was taken to help embed screening in daily practice [11, 12].

To facilitate ongoing training recording of scores was included in the flowsheet for all patients on the clinical information management system (CIMS) (IntelliSpace Critical Care & Anesthesia Information System (ICCA), Philips, United Kingdom) prior to this threshold being achieved. Screening was launched as formal policy on WDAD 11th March 2020.

### Participants

#### Inclusion criteria

Patients who met the criteria of the validated population for SOS-PD screening were included. This was all admissions ≥ 48 h, ≥ 3 months of age, who required invasive MV between 11 March 2019 and 12th March 2021. Encounters were divided into two cohorts; Cohort 1 before the official launch of delirium screening as standard of care: 11th March 2019-11th March 2020, and Cohort 2 after the official launch of delirium screening as standard of care: 12th March 2020–12th March 2021. Data were collected from day of admission until day of discharge or day 28 of PICU admission, whichever was sooner. Patients who never underwent invasive MV, were admitted < 48 h or with a history of head injury, neurological complications, comatosed state, deeply sedated or receiving continuous neuromuscular blocking agent infusions, not responding to stimuli and < 3 months of age were excluded.

#### Sample size

A formal sample size calculation was not carried out. The study site has approximately 1000 annual admissions to be screened for eligibility. All eligible patients during the study period were to be included to minimise risk of selection bias when sampling from the study population. The study period was limited to one year before and after implementation of screening to limit the effect of temporal changes in practices which may occur as time progresses.

#### Outcome measures

Primary outcomes were pharmacotherapy usage as measured by, percentage of patients receiving, dose per weight per day of use (dose/kg/day) and duration of use of: opioids, benzodiazepines, alpha agonists, sedating antihistamines and chloral hydrate.

Secondary outcomes included: PICU length of stay (LOS), duration of invasive MV, pain (NRS ≥ 5) and withdrawal (SOS ≥ 4) [13], time spent with COMFORT-B score between 11 and 16 [14], delirium screening compliance (% predicted scores), percentage with delirium detected, co-morbid conditions (as defined by Paediatric Complex Chronic Conditions) [19].

### Data sources

Cardiopulmonary bypass time was extracted from the National Institute for Cardiovascular Outcomes Research (NICOR) database [20]. Other data, unless specified otherwise, were extracted from ICCA with assistance from the PICU data manager.

### Statistical methods

Data were summarized using frequencies and percentages for categorical variables. Continuous data are described by mean and standard deviation, with those displaying some evidence of skew described by median and interquartile range (IQR). Chi-squared tests for categorical variables and Wilcoxon-rank sum for continuous variables were used to explore potential differences between cohorts. Dose per kg per day of use was calculated by dividing the total dose per kg by the number of days of use. Two multivariable logistic regression models for the potential differences between cohorts were explored; model 1 included variables that were clinically relevant and showed evidence of a statistical difference between cohorts and model 2 adjusted for these and risk factors identified in published research [4, 10]. Statistical analysis was undertaken with Stata v18 (StataCorp. 2019. Stata Statistical Software: Release 16. College Station, TX: StataCorp LLC.) and *p*-values < 0.05 deemed as statistically significant.

## Results

Two thousand one hundred and thirty-four patient encounters were identified with 588 meeting the inclusion criteria (364 Cohort 1 pre introduction of delirium screening and 224 Cohort 2 Post delirium screening). Fifty six percent of cohort 1 and 55% cohort 2 were male. The median age was 13 months (IQR5-75) for cohort 1 and 22 months (IQR5-76) for cohort 2. Cohort 2 had a significantly reduced number of respiratory admissions (*p* < 0.05), increased vasoactive use (*p* < 0.05), reduced invasive mechanical ventilation time (*p* < 0.05) but no evidence of differences in median Paediatric Index of Mortality 3 (PIM-3) scores (*p* = 0.1554) (Table 1) [21]. Otherwise all remaining parameters were similar.

**Table 1:** Demographic and Characteristics of PICU patients included (n = 588)

Characteristic	Cohort 1 Pre delirium scoring	Cohort 2 Post delirium scoring	*P* value
n	364	224	
% Male	56	55	0.82
Age (months) median and IQR^*a*^	13 (5–75)	22 (5–76)	0.19
Weight (kg) Median (IQR)	9.4 (6.2–18.9)	11.2 (6.3–20.7)	0.16
*Primary reason for admission*			
Cardiac	95 (26%)	58 (26%)	0.96
Cardio-Thoracic Surgery	119 (33%)	116 (52%)	< 0.05
Haematology/Oncology	7 (2%)	0 (0%)	< 0.05
Infection/Sepsis/Shock	22 (6%)	7 (3%)	0.11
Respiratory	63 (17%)	12 (5%)	< 0.05
Status Epilepticus	18 (5%)	12 (5%)	0.83
General Surgery	20 (5.5%)	13 (6%)	0.87
Other	20 (7%)	6 (3%)	0.11
*PIM3*^*b*^* Score*			
< 1%	150 (41%)	110 (49%)	0.06
1–5%	173 (48%)	102 (46%)	0.64
5–15%	38 (10%)	6 (2.8%)	< 0.05
15–30%	2 (0.6%)	5 (2%)	0.08
> 30%	1 (0.3%)	1 (0.5%)	0.62
PIM3 Score Median (IQR)	0.0120 (0.0076- 0.0271407)	0.0100 (0.0078–0.0213)	0.16
Survival to PICU^c^ discharge	349 (96%)	224 (100%)	< 0.05
Cardiothoracic surgery patients only	119	116	
*(% of CTS*^*d*^* patients)*			
Use of CPB^e^	102 (86%)	106 (91%)	< 0.05
Non Bypass	9 (7.6%)	2 (1.7%)	0.14
Other including hybrid, cardiac catheter procedures	8 (6.7%)	8 (6.9%)	0.32
Developmental delay	14 (3.8%)	6 (2.7%)	0.45
Vasoactives^f^	271 (74%)	193 (86%)	< 0.05
Unplanned Extubations	0 (0%)	2 (0.9%)	0.07
ECLS^f^	9 (2.5%)	4 (1.8%)	0.58

^a^ Interquartile range

^b^ Pediatric Index of Mortality-3

^c^ PICU = Pediatric Intensive Care Unit

^d^ CTS Cardiothoracic

^e^ CPB Cardiopulmonary bypass

^f^ Vasoactives as defined by PICANet include dobutamine, dopamine, adrenaline, noradrenaline, vasopressin and milrinone

^g^ Extracorporeal life support

n, number who received

One hundred and fourteen (51%) of the eligible patients from cohort 2 had at least one SOS-PD score completed during their admission. No patient had 100% compliance with screening. Of these 114 screened patients, 23 (20%) had a positive PD score of ≥ 4. Median day of detection of delirium was day 5 (IQR 2–8).

### Unadjusted analysis

*Opioid use* There was a reduction in opioid total dose and duration of use for cohort two, with, morphine infusion dose/kg/day 18% lower (*p* < 0.05) and enteral morphine doses mg/kg/day 15% lower (*p* < 0.05) (Table 2).

**Table 2:**
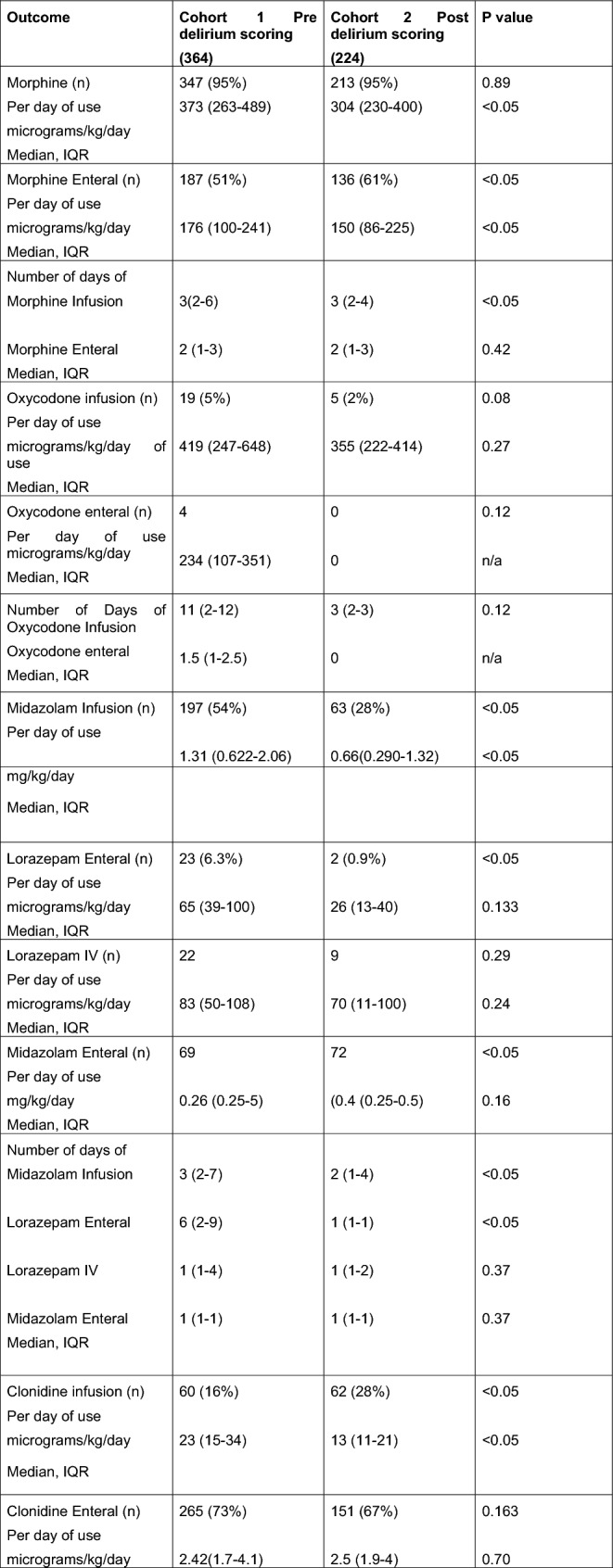

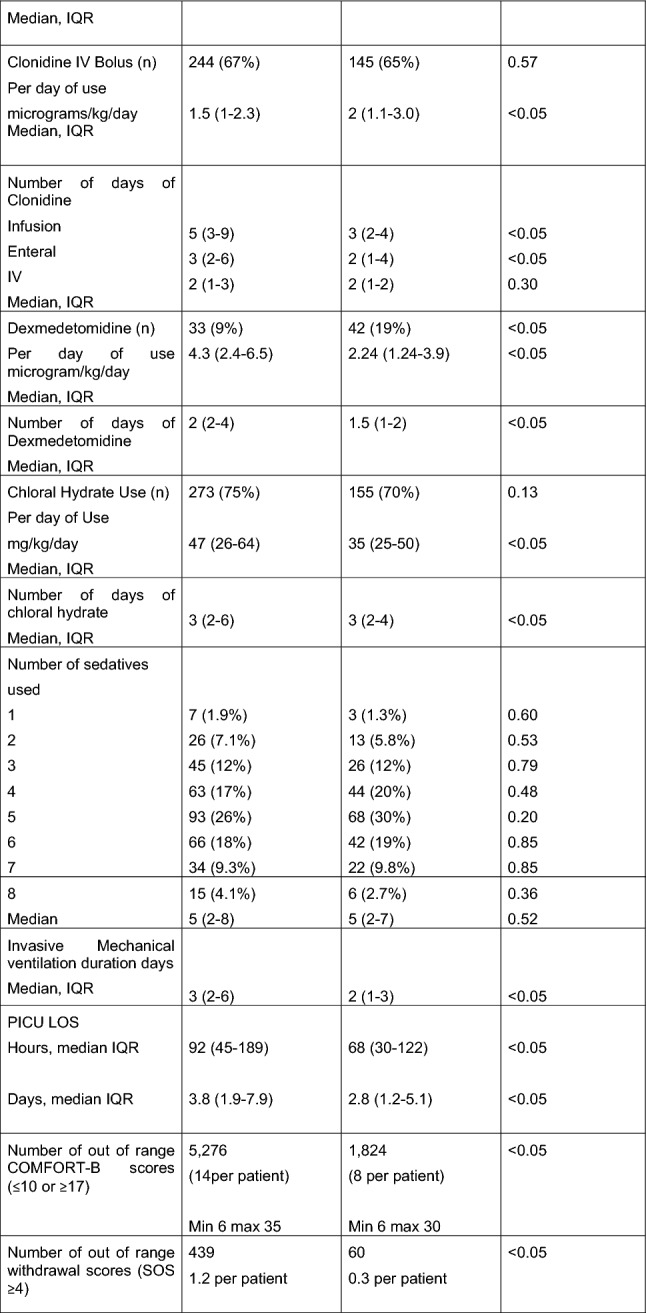
Unadjusted Outcomes and Associations of PICU patients included in the study (n = 588)

n, number who received; PICU, Paediatric Intensive Care Unit; IQR, Interquartile range; COMFORT-B, COMFORT Behavioural Score; SOS, Sophia Observation Withdrawal Score; IQR, Interquartile Range; IV, Intravenous

*Benzodiazepines* The number of patients who received midazolam infusions was reduced by 50% (*p* < 0.05) for cohort 2, with those who received daily doses half that in cohort 1 (*p* < 0.05). Where midazolam infusions were used the median number of days was one day less after delirium screening (3 days IQR 2–7, 2 days IQR 1–4 *p* < 0.05). The number of patients who received enteral lorazepam reduced by 90% (*p* < 0.05) for cohort 2 with no change in intravenous use of lorazepam or dose/kg/day of use (Table 2).

*Alpha agonists* Clonidine infusions were used more widely in cohort 2 (*p* < 0.05) however patients had a lower median daily dose (*p* < 0.05). The median dose/weight/day of clonidine intravenous (IV) bolus use was increased after delirium screening (cohort 2) from 1.5 µg/kg/day (IQR1-2) to 2 µg/kg/day (IQR 1–3) *p* < 0.05. The number of days of use of clonidine infusions, and enteral was reduced after delirium screening (cohort 2) (*p* < 0.05). Dexmedetomidine use increased (*p* < 0.05) after screening however dose per day of use was half for Cohort 2 compared to Cohort 1 (*p* < 0.05) and the number of days of use reduced from 2 (2–4) to 1.5 (1–2) *p* < 0.05 (Table 2) after screening.

The number of patients who received chloral hydrate did not change, with 75% of patients receiving. However Cohort 2 had lower doses per day of use (*p* < 0.05). The overall number of sedatives per patient in both groups remained similar (median 5 *p* = 0.52).

### Adjusted analysis

Multivariable logistic regression identified differences between cohort 1 (before delirium screening) to cohort 2 (after delirium screening) in relation to an increased number of patients who received clonidine infusions (OR 2.28, 95% CI1.48–3.5, *p* < 0.05), dexmedetomidine infusions (OR 1.98, 95% CI 1.19–3.3, *p* < 0.05) and midazolam enteral (OR 1.72 95% CI 1.14–2.61, *p* < 0.05) after screening (Table 3). A decreased use of enteral lorazepam (OR 0.12 95% CI 0.03–0.57, *p* < 0.05) and midazolam infusions (OR 0.38 95% CI 0.26–0.57, *p* < 0.05) was observed in Cohort 2 (Table 3).

**Table 3:** Multivariable association and adjusted odds ratios for individual drugs administered in Cohort 2 vs Cohort 1

Days	Multivariable logistic regression Model 1*		Multivariable logistic regression Model 2**	
	OR (95% CI)	*p*-value	OR (95% CI)	*p*-value
Morphine infusion	0.64 (0.27–1.49)	0.303	0.76 (0.32–1.83)	0.56
Morphine enteral	1.12 (0.78–1.61)	0.546	1.29 (0.87–1.91)	0.20
Oxycodone infusion	0.45 (0.16–1.24)	0.124	0.36 (0.13–1.04)	0.06
Chloral	0.68 (0.46–1.01)	0.056	0.74 (0.46–1.18)	0.20
Antihistamine	0.56 (0.25–1.29)	0.174	0.57 (0.25–1.31)	0.189
Clonidine infusion	2.33 (1.52–3.57)	< 0.05	2.28 (1.48–3.50)	< 0.05
Clonidine enteral	0.79 (0.54–1.16)	0.233	0.87 (0.59–1.30)	0.51
Clonidine IV bolus	0.73 (0.50–1.06)	0.099	0.75 (0.51–1.10)	0.14
Dexmedetomidine	2.07 (1.25–3.43)	< 0.05	1.98 (1.19–3.30)	< 0.05
Lorazepam enteral	0.15 (0.03–0.64)	< 0.05	0.12 (0.03–0.57)	< 0.05
Lorazepam IV	0.80 (0.35–1.82)	0.592	0.63 (0.26–1.50)	0.29
Midazolam infusion	0.41 (0.28–0.60)	< 0.05	0.38 (0.26–0.57)	< 0.05
Midazolam enteral	1.62 (1.08–2.43)	< 0.05	1.72 (1.14–2.61)	< 0.05
Fentanyl infusion	0.38 (0.08–1.78)	0.219	0.38 (0.08–1.78)	0.22
Propofol	0.64 (0.33–1.24)	0.189	0.50 (0.25–1.02)	0.06

OR, Odds Ratio; CI, Confidence Interval

^*^Adjusted for Respiratory/Cardio-thoracic admission, Paediatric Index of Mortality-3, vasoactive-use

^**^Adjusted for Respiratory/Cardiao-thoracic admission, Paediatric Index of Mortality-3 Score, weight, vasoactive-use, gender

^$^Development delay and COMFORT-B score out of range omitted because of collinearity

Due to the increase in respiratory admissions and decrease in post-operative cardiothoracic admissions during the study period, statistical analysis was carried out in these sub-groups (Supplementary Tables 1 and 2). Sub-group analysis found consistent with overall results a move away from midazolam infusions in cardiothoracic patients for cohort 2 (12% reduction *p* < 0.05) and a reduction in the dose of morphine infusions used for respiratory patients (32% *p* < 0.05). Sub-group analysis for cardiothoracic patients showed statistically significant increased use of enteral midazolam (52% *p* < 0.05), clonidine infusions (180% *p* < 0.05), higher IV clonidine doses (33% *p* < 0.05), with a reduction in enteral morphine daily doses (28% *p* < 0.05) for cohort 2. Sub-group analysis of respiratory admissions showed lower doses of morphine infusion (405 µg/kg/day (IQR287-601) V 277 µg/kg/day (171–384) *p* < 0.05), and higher use of clonidine infusions (19% V 58% *p* < 0.05) for cohort 2. These figures are consistent with the overall trends. There was no evidence of a change to invasive MV days or PICU LOS in either sub-group.

**Fig. 1:**
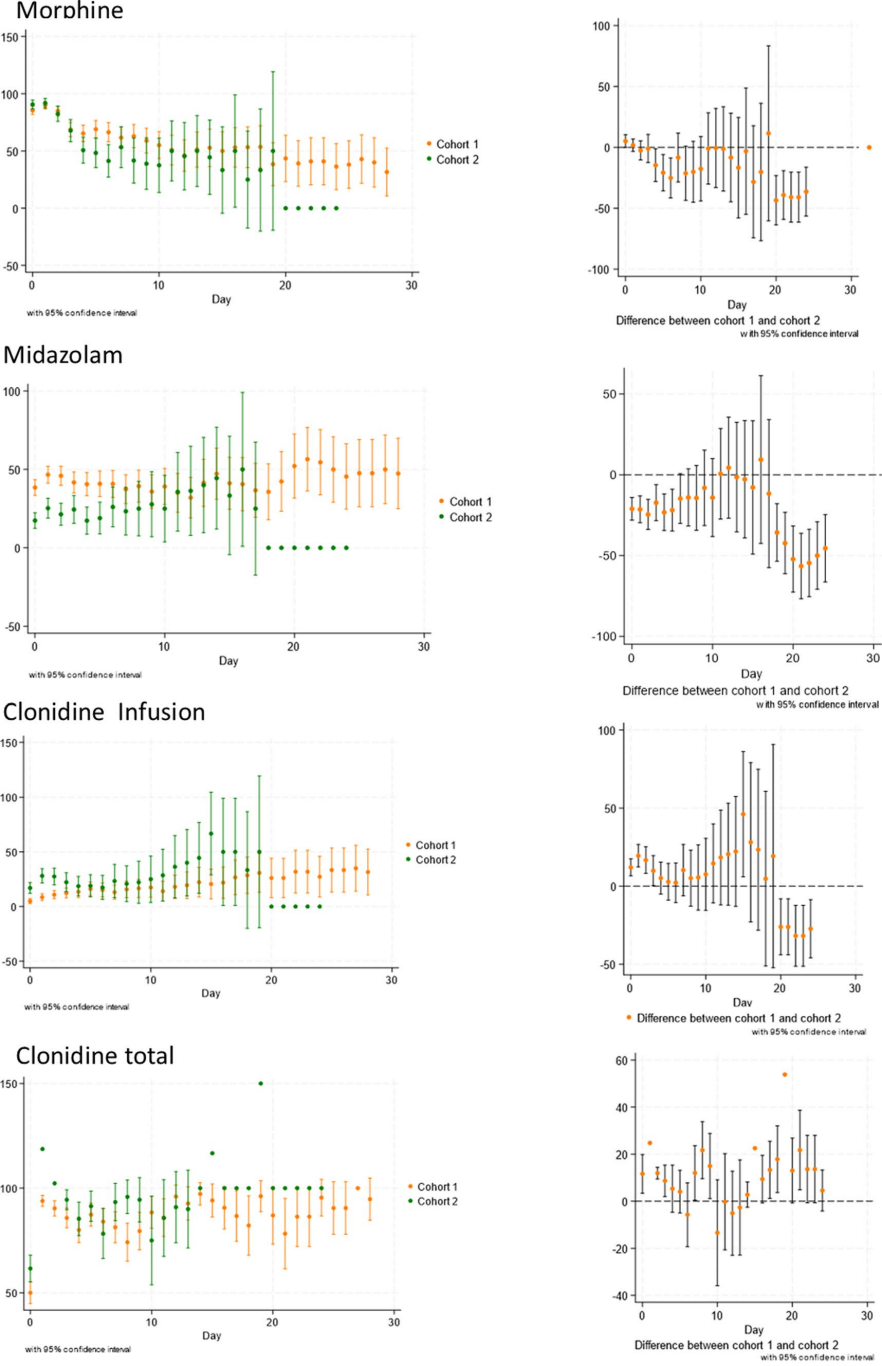
Percentage of patients using medication per day of admission for each cohort and difference in use between cohorts per day of admission

Figure 1 shows a change in prescribing practices from Day 1 of admission of a patient with more patients receiving clonidine infusions and less patients receiving midazolam infusions after screening commenced. There was evidence of a statistical difference for use of morphine infusions at day 5, use of midazolam infusions in the first 5 days and after day 18, clonidine infusion use in the first 3 days, and clonidine enteral use beyond day 20 between the cohorts. Interpretation of data beyond day 10 is difficult due to small patient numbers.

There was evidence of a statistically significant difference in survival to PICU discharge despite the non-significant PIM-3 scores.

## Discussion

The use of benzodiazepines, opioids and other sedatives are modifiable risk factors for the development of delirium [10, 22]. We believe that the introduction of a bundle of education resources, tools and protocol for paediatric delirium screening created awareness and a culture that resulted in changes to pharmacological management of MV patients in PICU. Whilst the primary goal when introducing delirium screening is that every child is screened and instances of PD identified, this study shows that even where screening rates remain low the introduction of screening can affect the management of patients. We believe this is an important lesson for those who are introducing delirium screening to their PICU. Ultimately the goal of all such interventions and tools are for better patient care, and we have shown the reduction in exposure to a modifiable risk factor for paediatric delirium development without affecting clinical outcomes.

The study period coincided with the SARS-CoV-2 pandemic, with lockdown measures announced for Ireland on March 12th 2020 [23]. The introduction of ‘lockdown’ measures has been shown to have decreased respiratory and post operative cardiothoracic admissions to the PICU [24]. However our figures show an increase in post-operative cardiothoracic admission rate [24]. The increase in use of enteral midazolam, which is part of a chest drain removal protocol and increase in vasoactive and dexmedetomidine use, that from May 2019 is approved for use in the prevention of post-operative junctional ectopic tachycardia (JET), is indicative of this increased proportion of cardiothoracic patients [25]. The reduction in invasive MV days and PICU LOS for Cohort 2 may be indicative of this changing demographic.

Sub-group analysis of the cardiothoracic cohort (Supplementary Table 2) shows no change to these variables indicating a change in prescribing practices did not influence these variables when a more homogenous population is studied.

The goal of sedation in respiratory patients is to sedate and analgise to tolerate ventilation, however in post-operative cardiothoracic patients the goal is to wake, wean, extubate mobilse and discharge. In the sub-group analysis the use of midazolam infusions was unchanged in respiratory patients but did reduce in cardiothoracic surgical patients, consistent with the overall trends. Generally morphine infusion use decreased in both sub-populations (32% in respiratory *p* < 0.05, 7% cardiothoracic *p* > 0.05). The number of out of range COMFORT-B scores per patient were reduced after delirium screening and the number of SOS scores ≥ 4, indicating IWS, was reduced for cohort 2 (*p* < 0.05). This is likely a factor of the reduction in benzodiazepine usage. We cannot exclude that the difference in admission demographic influenced the change in prescribing practices.

During the study period a study on the pharmacokinetics of clonidine was published, and this along with the general move away from benzodiazepines may have had an effect on the prescribing pattern of clonidine, however despite this work we did not identify higher doses of clonidine infusions that would have been expected [26]. Local overarching guidelines for analgesia and sedation, as well as the delirium screening guidelines did not change over the study period. This gives some confidence that changes in outcomes are unlikely due to be the result of other changes in practice over the two year study period.

The SOS-PD score was validated by Ista et al. in a multisite study with investigators trained in each site [12]. The team in CHI Crumlin had not been trained directly by these investigators but used the validation tool, accompanying instructions available in the published papers and resources provided publicly to develop our intervention bundle [11, 12, 18]. This could lead to some subjectivity in the application of the score by staff in CHI as opposed to in the validation sites. However, this method of introducing new practices is not unique to our intervention or site. The use of restraints has been identified as a contributory factor towards the development of paediatric delirium [4]. However, this information is not currently universally collected in the PICU CIMS, and so could not be evaluated with the modifiable risk factor parameters.

This research provides important implications for practice. Previous work has suggested four of the seven risk factors for paediatric delirium are modifiable [10]. Pharmacists and Clinicians should introduce formal paediatric delirium screening within their units to actively identify and manage exposure to modifiable risk factors at patient review. Having a formal guideline, screening process and associated education can increase environmental awareness of the complication of paediatric delirium and potentially reduce negative sequelae for critically ill children. Although observational in nature, our study adds to the body of work showing sedation exposure can be reduced without increasing patient discomfort or causing harm. Future research in the form of interventional cluster RCTs should evaluate the role of PD bundles in reducing exposure to modifiable risk factors such as analgesia and sedation.

## Conclusion

We found a change in prescribing practices of analgesia and sedation after the introduction of a paediatric delirium screening tool and associated education. This change did not negatively impact clinical outcomes such as duration of mechanical ventilation and length of stay. Due to the impact of the COVID-19 pandemic on admission type these findings should be further evaluated in future multi-site interventional studies.

## Supplementary Information

Below is the link to the electronic supplementary material.

Supplementary file1 (PDF 293 KB)

Supplementary file2 (DOCX 514 KB)

Supplementary file3 (DOCX 32 KB)

Supplementary file4 (DOCX 31 KB)
