# Supplementary material for: Delirium in critically ill children: a retrospective pre- and post-cohort study on the introduction of delirium screening in a paediatric intensive care unit
Source: Int J Clin Pharm. 2025 May 7;47(3):844–53. doi: 10.1007/s11096-025-01887-2 (PMC12125132; doi:10.1007/s11096-025-01887-2)
Supplement: Supplementary file 1 — Supplementary file1 (PDF 293 KB) [file 11096_2025_1887_MOESM1_ESM.pdf]

SOS Paediatric Delirium Score

Score ≥ 4

Identify potential causes and treat- BRAINMAPS*

B	• Bring oxygen (e.g hypoxemia, anemia).
R	• Remove or wean drugs that cause
A	• Atmosphere (e.g. bright lights, loud noises, unfamilar people).
I	• Inflamation, infection and immobilisation.
N	• New organ dysfunction.
M	• Metabolic disturbance (e.g acidosis, electrolyte imbalance).
A	• Awake (e.g sleep wake disturbances)
P	• Pain
S	• Sedation

Score < 4

Reassessment on next shift or if concerned patient may be delirious.

Initiate strategies to reduce delirium

Sleep and Environment

- Increase light exposure during daytime and create dark environment at night to encourage natural sleep-wake cycle.
- Minimise night time interventions
- Minimise napping during the day.
- Relaxing music before bed.
- Noise reduction (consider use of ear plugs/masks).
- Address sensory limitations e.g. glasses, hearing aids or use of communication boards.

Parental Engagement

- Create familiar environment- music, books and toys from home.
- Encourage parents to speak to their child, reassure them and re- orientate them to the environment.
- Encourage parents to participate in their child's care (as safety allows) - feeding, washing and dressing and changing nappies.
- Create a daily schedule with parents to emulate the home schedule.
- Hypoactive delirium- increase social interaction with family members.

Early Mobility

- Spectrum of passive and active activities are considered early mobility.
- Position in developmentally appropriate positions- hands together in midline.
- Limit TV/Screen time and promote developmentally appropriate activities e.g. toys jigsaws, blocks. Consider referral to play therapists.
- Minimal restraints (if possible).
- Team to consider out of bed to chair/parents arms +/- ambulation if appropriate.
- Consider referral to Physiotherapy/Occupational therapy.

Pharmacological

- Last line.
- Treat Pain – simple analgesia eg paracetamol and/or ibuprofen.
- Avoid and discontinue drugs that reinforce delirium (if possible) e.g. benzodiazepines such as midazolam/lorazepam, and anticholinergics such as oxybutynin, ipratropium and hyoscine.
- 1st Line Clonidine (high dose). Consider risperidone and/or haloperidol as last resort

If no improvement or significantly high score consider referral to psychiatry

Remember
Prevention is more effective than treatment. Do not wait until delirium develops to enact the above strategies – minimise exposure to sedation agents if possible.

*BRAINMAPS adapted from: Smith HA, Brink E, Fuchs DC, et al. 2013. Pediatric delirium: Monitoring and management in the pediatric intensive care unit. *Pediatr Clin North Am*. 60(3):741–760
