# Supplementary figures and images for: Delirium in critically ill children: a retrospective pre- and post-cohort study on the introduction of delirium screening in a paediatric intensive care unit
Source: Int J Clin Pharm. 2025 May 7;47(3):844–53. doi: 10.1007/s11096-025-01887-2 (PMC12125132; doi:10.1007/s11096-025-01887-2)

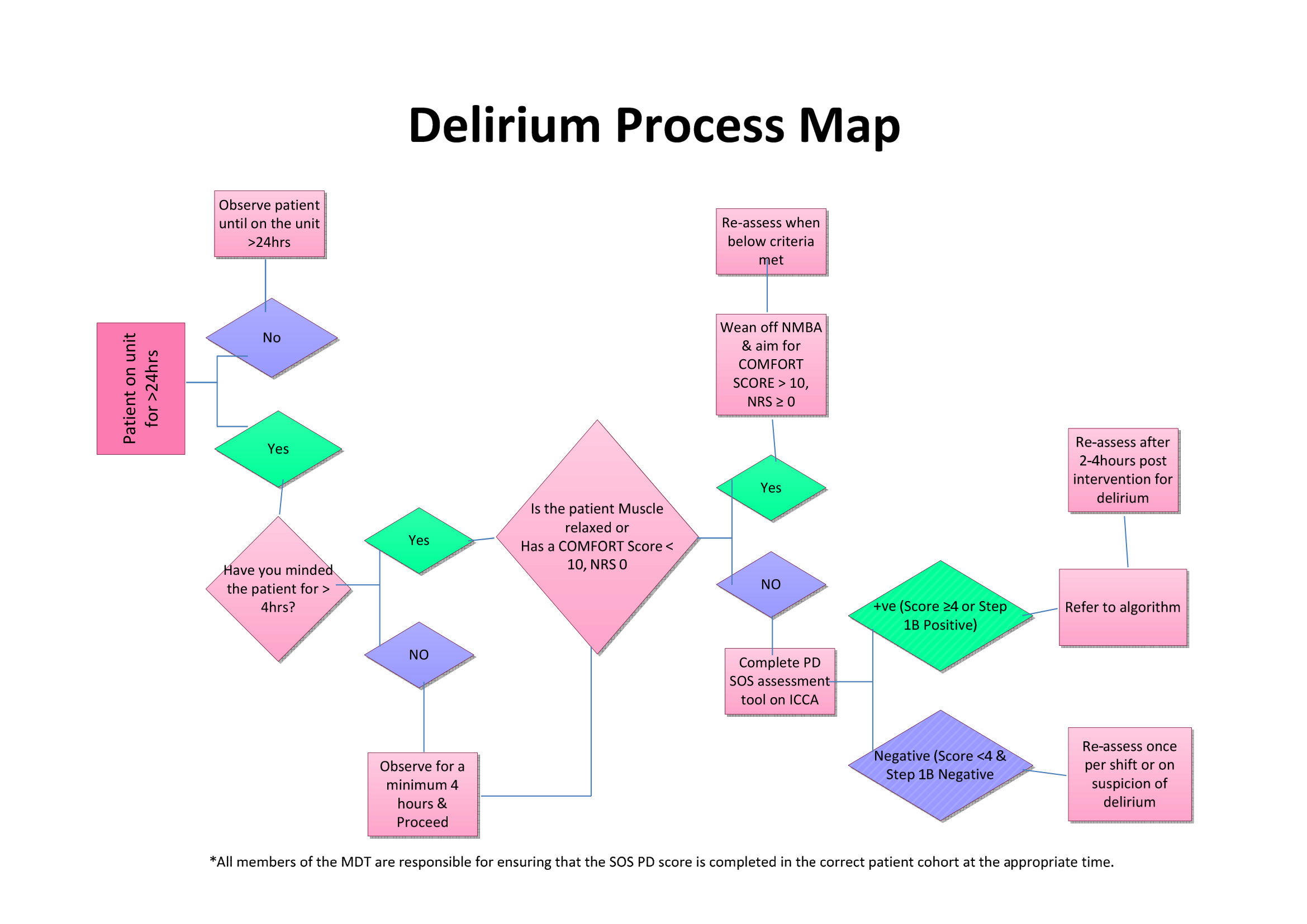


Figure 1: Delirium Process Map PICU CHI Crumlin

Supplement: Supplementary file 2 — Supplementary file2 (DOCX 514 KB) [file 11096_2025_1887_MOESM2_ESM.docx]
