# Supplementary material for: Delirium in critically ill children: a retrospective pre- and post-cohort study on the introduction of delirium screening in a paediatric intensive care unit
Source: Int J Clin Pharm. 2025 May 7;47(3):844–53. doi: 10.1007/s11096-025-01887-2 (PMC12125132; doi:10.1007/s11096-025-01887-2)
Supplement: Supplementary file 3 — Supplementary file3 (DOCX 32 KB) [file 11096_2025_1887_MOESM3_ESM.docx]

# Delirium in critically ill children: A retrospective pre- and post-study on the introduction of delirium screening in a paediatric intensive care unit.

International Journal of Clinical Pharmacy,

**Supplementary Table 1**

Unadjusted Outcomes and Associations for respiratory patients only

| **Outcome** | **Cohort 1 Pre delirium scoring**  **(63)** | **Cohort 2 Post delirium scoring**  **(12)** | **P value** |
| --- | --- | --- | --- |
| Morphine (n*^a^*)  Per day of use  micrograms/kg/day  Median, IQR*^b^* | 54 (86%)  405 (287-601) | 12 (100%)  277(171-384) | 0.16  <0.05 |
| Morphine Enteral (n*^a^*)  Per day of use  micrograms/kg/day  Median, IQR*^b^* | 20 (32%)  150(98-214) | 5 (42%)  80(59-120) | 0.50  0.16 |
| Number of days of  Morphine Infusion  Morphine Enteral  Median, IQR*^b^* | 5.5(3-8)    3 (2-4) | 4.5 (3-8)    2 (1-2) | 0.63  0.05 |
| Oxycodone infusion (n*^a^*)  Per day of use  micrograms/kg/day of use  Median, IQR*^b^* | 3 (4.8%)  720 (72-841) | 0(0%)  n/a | 0.44  n/a |
| Oxycodone enteral (n*^a^*)  Per day of use  micrograms/kg/day  Median, IQR*^b^* | 1  95 (95-95) | 0 | n/a |
| Number of days of  Oxycodone Infusion  Oxycodone enteral  Median, IQR*^b^* | 11 (4-11)  1 (1-1) | 0  0 | n/a  n/a |
| Midazolam Infusion (n*^a^*)  Per day of use  mg/kg/day  Median, IQR*^b^* | 45 (71%)  1.34 (0.6102-2.06) | 8 (67%)  1.30(0.307-1.76) | 0.74  0.47 |
| Lorazepam Enteral (n*^a^*)  Per day of use  micrograms/kg/day  Median, IQR*^b^* | 4 (6.3%)  100 (75-189) | 0  n/a | 0.37  n/a |
| Lorazepam IV (n*^a^*)  Per day of use  micrograms/kg/day  Median, IQR*^b^* | 4 (6.3)  108 (91-125) | 1 (8%)  70 ((70-70) | 0.80  0.16 |
| Midazolam Enteral (n*^a^*)  Per day of use  mg/kg/day  Median, IQR*^b^* | 2  0.311 (0.25-0.375) | 0  n/a | 0.53  n/a |
| Number of days  Midazolam Infusion Use  Lorazepam Enteral  Lorazepam IV*^c^*  Midazolam Enteral  Median, IQR*^b^* | 4 (2-7)  4 (28)  1 (1-2)  1 (1-1) | 4 (3-6)  n/a  2 (2-2)  n/a | 0.91  n/a  0.43  n/a |
| Midazolam Enteral (n*^a^*)  per day used mg/kg/day | 2  0.311 (0.25-0.375) | 0  n/a | 0.53  n/a |
| Clonidine infusion (n*^a^*)  Per day of use  micrograms/kg/day  Median, IQR*^b^* | 12 (19%)  22 (13-28) | 7 (58%)  16 (8-25) | <0.05  0.27 |
| Clonidine Enteral (n*^a^*)  Per day of use  micrograms/kg/day  Median, IQR*^b^* | 59 (94%)  3.3(1.8-4.9) | 10 (83%)  3.9 (1.4-9.7) | 0.23  0.44 |
| Clonidine IV*^c^* Bolus (n*^a^*)  Per day of use  micrograms/kg/day  Median, IQR*^b^* | 27 (43%)  2 (1-3.5) | 5 (42%)  2 (1-3) | 0.94  0.86 |
| Number of days of Clonidine  Infusion  Enteral  IV*^c^*  Median, IQR | 4.5 (2.5-6)  4 (2-7)  2 (1-3) | 3 (2-7)  3 (2-5)  2 (1-3) | 0.73  0.39  0.81 |
| Dexmedetomidine (n*^a^*)  Per day of use microgram/kg/day  Median, IQR*^b^* | 2(3%)  10 (6-15) | 1 (8%)  12 (12-12) | 0.40  1 |
| Number of days of dexmedetomidine  Median, IQR*^b^* | 3 (2-44) | 4 (4-4) | 0.48 |
| Chloral Hydrate Use (n*^a^*)  Per day of Use  mg/kg/day  Median, IQR*^b^* | 56 (89%)  54 (38-77) | 9 (75%)  45 (33-86) | 0.35  1 |
| Number of days of chloral hydrate  Median, IQR*^b^* | 4.5 (3-6.5) | 4 (2-5) | 0.37 |
| Number of sedatives Used  1  2  3  4  5  6  7  8  9  10  Median, IQR*^b^* | 2(3.2%)  4 (6.5%)  5 (7.9%)  14 (22%)  18 (29%)  10 (16%)  6 (9.5%)  2 (3.2%)  1 (1.6%)  1 (1.6%)  5 (4-6) | 0 (0%)  1 (8.3%)  1 (8.3%)  1 (8.3%)  4 (33%)  2 (17%)  3 (25%)  0 (0%)  0 (0%)  0 (0%)  5 (6-7) | 0.53  0.80  0.96  0.27  0.74  0.95  0.13  0.53  0.66  0.66  0.44 |
| Invasive Mechanical ventilation duration days  Median, IQR*^b^* | 6 (4-9) | 4.5 (3.5-9) | 0.57 |
| PICU*^d^* LOS*^e^*  Hours, median IQR*^b^*  Days, median IQR*^b^* | 173 (91-253)  7.2 (3.8-10.6) | 119 (95-248)  4.9 (4.0-10.3) | 0.55  0.55 |

a number who received, b interquartile range, c Intravenous, d Pediatric Intensive Care Unit, e length of stay
