# Supplementary material for: Delirium in critically ill children: a retrospective pre- and post-cohort study on the introduction of delirium screening in a paediatric intensive care unit
Source: Int J Clin Pharm. 2025 May 7;47(3):844–53. doi: 10.1007/s11096-025-01887-2 (PMC12125132; doi:10.1007/s11096-025-01887-2)
Supplement: Supplementary file 4 — Supplementary file4 (DOCX 31 KB) [file 11096_2025_1887_MOESM4_ESM.docx]

# Delirium in critically ill children: A retrospective pre- and post-study on the introduction of delirium screening in a paediatric intensive care unit.

International Journal of Clinical Pharmacy,

**Supplementary Table 2**

Unadjusted Outcomes and Associations for cardiothoracic patients only

| **Outcome** | **Cohort 1 Pre delirium scoring**  **(119)** | **Cohort 2 Post delirium scoring**  **(116)** | **P value** |
| --- | --- | --- | --- |
| Morphine (n*^a^*)  Per day of use  micrograms/kg/day  Median, IQR*^b^* | 118 (99%)  326 (237-406) | 114 (99%)  303 (229-379) | 0.55  0.30 |
| Morphine Enteral (n*^a^*)  Per day of use  micrograms/kg/day  Median, IQR*^b^* | 83(70%)  194 (116-240) | 86 (74%)  139 (87-220) | 0.45  <0.05 |
| Number of days of  Morphine Infusion  Morphine Enteral  (Median, IQR*^b^*) | 3(2-4)  2 (1-2) | 3 (2-4)  2 (1-3) | 0.98  0.53 |
| Oxycodone infusion (n*^a^*)  Per day of use  micrograms/kg/day of use  Median, IQR*^b^* | 3 (2.5%)  234 (190-247) | 2 (1.7%)  384 (355-414) | 0.67  0.08 |
| Oxycodone Enteral (n)  Per day of use  micrograms/kg/day  Median, IQR*^b^* | 1 (0.8%)  354 (354-354) | 0  n/a | n/a |
| Number of days of Oxycodone  Infusion  Oxycodone enteral  Median, IQR*^b^* | 2 (1-3)  2 (2-2) | 4.5 (3-6)  n/a | 0.14  n/a |
| Midazolam Infusion (n*^a^*)  Per day of use  mg/kg/day  Median, IQR*^b^* | 33 (28%)  0.91 (0.297-1.37) | 18 (16%)  0.61(0.067-1.05) | <0.05  0.35 |
| Lorazepam Enteral (n*^a^*)  Per day of use  micrograms/kg/day  Median, IQR*^b^* | 2 (1.7%)  74 (31-117) | 1 (0.86%)  40 (40-40) | 0.58  1 |
| Lorazepam IV*^c^* (n*^a^*)  Per day of use  micrograms/kg/day  Median, IQR*^b^* | 0  n/a | 2 (1.7%)  55 (11-98) | 0.15  n/a |
| Midazolam Enteral (n*^a^*)  Per day of use  mg/kg/day  Median, IQR*^b^* | 32 (27%)  0.29 (0.25-0.5) | 47 (41%)  0.26 (0.25-0.5) | <0.05  0.48 |
| Number of days  Midazolam Infusion Use  Midazolam Enteral  Lorazepam Enteral  Lorazepam IV*^c^* | 3 (2-4)  1 (1-1)  8.5 (7-10)  n/a | 2 (2-4)  1 (1-1)  1 (1-1)  1.5 (1-2) | 0.60  0.71  0.22  n/a |
| Clonidine infusion use (n*^a^*)  Per day of use  micrograms/kg/day  Median, IQR*^b^* | 10 (8.4%)  16 (8.7-21) | 28 (24%)  13 (7.9-14) | <0.05  0.32 |
| Clonidine Enteral (n*^a^*)  Per day of use  micrograms/kg/day  Median, IQR*^b^* | 88 (74%)  2(1.5-3) | 88 (76%)  2.6 (1.9-4) | 0.74  0.08 |
| Clonidine IV*^c^* Bolus (n*^a^*)  Per day of use  micrograms/kg/day  Median, IQR*^b^* | 85(71%)  1.5 (1-2) | 81 (70%)  2 (1-3) | 0.79  <0.05 |
| Number of days of clonidine  Infusion  Enteral  IV*^c^*  Median, IQR*^b^* | 4 (2-6)  2 (2-4)  2 (1-2) | 2 (2-3)  2 (1-4)  2 (1-2) | 0.31  0.34  <0.05 |
| Dexmedetomidine (n*^a^*)  Per day of use microgram/kg/day  Median, IQR*^b^* | 16 (13%)  3.4 (1.5-4.7) | 19 (16%)  2.2 (1.1-4.3) | 0.53  0.29 |
| Number of days of Dexmedetomidine  Median, IQR*^b^* | 2 (1-2) | 1 (1-2) | 0.14 |
| Chloral Hydrate Use (n*^a^*)  Per day of Use  mg/kg/day  Median, IQR*^b^* | 97 (82%)  41 (25-59) | 93 (60%)  38 (25-53) | 0.79  0.36 |
| Number of days of chloral hydrate | 3 (2-5) | 3 (2-4) | 0.89 |
| Number of sedatives Used  1  2  3  4  5  6  7  8  9  10  Median, IQR*^b^* | 1 (0.8%)  8 (6.7%)  11 (9.2%)  26 (22%)  33 (28%)  28 (24%)  9 (7.6%)  2 (1.7%)  0 (0%)  1 (0.8%)  5 (4-6) | 0 (0%)  2 (1.7%)  8 (6.9%)  29 (25%)  36 (31%)  28 (24%)  10 (8.6%)  3 (2.6%)  0 (0%)  0 (0%)  5 (4-6) | 0.322  0.058  0.509  0.568  0.578  0.913  0.766  0.631  n/a  0.322  0.3232 |
| Invasive Mechanical ventilation duration days  Median IQR*^b^* | 2 (1-3) | 2 (1-2.5) | 0.5327 |
| PICU*^d^* LOS*^e^*  Hours, median IQR*^b^*  Days, median IQR*^b^* | 59 (25-97)  2.5 (1.1-4.1) | 58 (29-97)  2.4 (1.2-4.0) | 0.8969  0.8969 |

a number who received, b interquartile range, c Intravenous, d Pediatric Intensive Care Unit, e length of stay
